# Menopausal Symptoms, Perceived Workplace Openness and Work Productivity Among Japanese Women: Baseline Findings from a Large-Scale Cohort Study

**DOI:** 10.3390/ijerph23020186

**Published:** 2026-01-31

**Authors:** Makiko Arima, Yoshikuni Edagawa, Kohta Suzuki, Chikako Kawahara, Nahoko Shirato, Yoshie Miwa, Miki Izumi

**Affiliations:** 1 School of Medicine, Showa Medial University, Tokyo 142-8777, Japan; 2 Graduate School of Technology Management, Ritsumeikan University, Osaka 567-8570, Japan; 3 School of Medicine, Aichi Medical University, Nagakute 480-1195, Japan; 4 Simulation Center, Teikyo University, Tokyo 173-8605, Japan; 5 Women’s Healthcare Awareness & Menopause Network Society, Shinjuku-ku, Tokyo 160-0008, Japan

**Keywords:** menopause, work productivity, psychological symptoms, workplace openness, Japan

## Abstract

**Highlights:**

**Public health relevance—How does this work relate to a public health issue?**

- Menopausal symptoms are highly prevalent among midlife working women and can become an equity concern in occupational health.
- Low psychological safety in the workplace can exacerbate the effects of menopausal symptoms on work functioning.

**Public health significance—Why is this work of significance to public health?**

- Menopause-related psychological and physical symptoms can impair concentration, communication, and decision-making at work.
- Supportive organizational climates may help sustain women’s health and employment during midlife.

**Public health implications—What are the key implications or messages for practitioners, policy makers and/or researchers in public health?**

- Integrating occupational health professionals and fostering psychologically safe work environments can support women’s well-being and help maintain productivity during the menopausal transition.
- Organizational support for menopausal health aligns with public health priorities, including disease prevention, mental health promotion, and reducing health disparities among working women.

**Abstract:**

This study analyzed baseline data from a six-month longitudinal cohort to describe the demographic, occupational, and symptom profiles of Japanese working women and to examine associations between menopausal symptoms, workplace openness, and work productivity. A total of 4000 women aged 40–60 years completed the Simplified Menopause Index (SMI), a commonly used measure in Japan to assess menopausal symptoms, and the validated Health and Work Performance Questionnaire (HPQ) to assess self-rated work productivity. Multiple regression analyses were conducted, adjusting for demographic and occupational covariates. Psychological symptoms showed the strongest negative association with work productivity (β = −0.186, *p* < 0.001), while vasomotor symptoms showed a small positive coefficient (β = 0.054, *p* = 0.007). Somatic symptoms were not significant (β = −0.033, *p* = 0.121). Lower perceived workplace openness was associated with lower productivity (β = −0.149, *p* < 0.001), such that employees who felt uncomfortable or unsure about discussing health concerns reported lower productivity. Higher educational attainment, longer working hours, and longer years of service were also associated with higher productivity. These findings indicate that psychological and physical symptoms are associated with lower work functioning during midlife, while supportive organizational environments appear to be relevant in this context. These cross-sectional findings provide a foundation for future longitudinal analyses and highlight the potential relevance of workplaces that promote open health communication.

## 1. Introduction

In Japan, about one-quarter of the 30.6 million working women are aged 45–55 years, and their labor force participation rate exceeds 70% [1]. This age group coincides with the menopausal transition. Menopause refers to the approximately 10-year period surrounding the cessation of ovarian function, typically occurring between ages 45 and 55 [2]. Among Japanese women, the average age at menopause is reported to be 50.5 years [3]. During this period, declining estrogen levels can lead to various vasomotor, psychological, and somatic symptoms. Previous studies have shown that around 80% of midlife women experience menopausal symptoms [2,4], such as hot flashes, fatigue, mood changes, reduced concentration, and depressive feelings, which may lower productivity and impede career advancement [5]. Many women struggle to balance work and daily life due to these symptoms [6].

Furthermore, menopause and other female-specific health concerns often remain difficult to discuss openly in Japanese workplaces, where formal support systems are still limited. Reports indicate that women frequently find it challenging to talk about their symptoms at work or to receive understanding from colleagues and supervisors [7,8]. Consequently, the menopausal transition represents a critical phase for women’s health and work life. As more midlife women continue to participate in the workforce, understanding the impact of menopausal symptoms on productivity has become an important social and occupational issue. Although previous studies have found that more severe symptoms are associated with lower presenteeism and productivity [6], many were constrained by small sample sizes or insufficient consideration of workplace context [7,9]. Large-scale studies in Japan examining the combined impact of menopausal symptoms and workplace environment on work productivity remain scarce.

While a growing body of research has examined the association between menopausal symptoms and work productivity, including studies conducted in Japan, several important gaps remain. First, most prior studies have focused primarily on symptom severity, with limited attention to workplace-related contextual factors that may coexist with menopausal symptoms in shaping work productivity. Second, few studies have simultaneously examined multiple symptom domains alongside organizational characteristics within a single analytical framework.

Accordingly, the present study aimed to describe cross-sectional associations between menopausal symptom domains, workplace openness, and self-rated work productivity, based on baseline data from a nationwide cohort of midlife Japanese working women. Using data from a large-scale baseline survey, menopausal symptoms were assessed using the Simplified Menopause Index (SMI). The SMI was analyzed both as a total score and across its three symptom domains—vasomotor, psychological, and somatic—to examine their differential associations with productivity in the context of health and productivity management. All three domains were simultaneously included in the multivariable model to compare their relative associations with work productivity.

## 2. Materials and Methods

### 2.1. Study Design and Participants

This cross-sectional analysis is based on baseline data collected as part of a six-month longitudinal cohort survey of 4000 Japanese working women. The primary objective of the present analysis was to describe participants’ demographic, occupational, and health-related characteristics at baseline and to explore the associations between menopausal symptoms, workplace openness, and self-rated work productivity.

**Figure 1:**
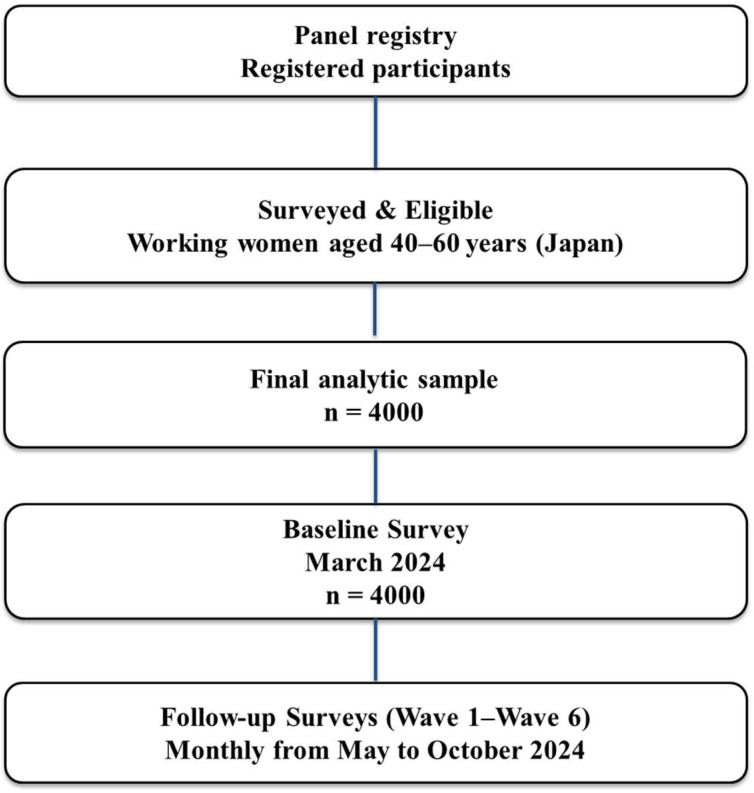
Study design and participant flow. A total of 4000 working women aged 40–60 years were recruited from a national online research panel and completed the baseline survey in March 2024. Six additional survey waves (Waves 1–6) were scheduled between May and October 2024; the present study reports findings from the baseline survey only.

Participants were recruited through a nationwide online research panel and represented a broad range of occupations, industries, and employment types. Eligible participants were employed women aged 40–60 years who completed all relevant questionnaire items. Figure 1 illustrates the recruitment and screening process, resulting in a final sample of 4000 respondents who completed the baseline survey. The questionnaire assessed demographic characteristics, employment status, menopausal symptoms, work productivity, and workplace openness regarding health-related discussions. All participants provided informed consent prior to participation. As is common with online panel surveys, a conventional response rate could not be calculated because invitations were sent to a large pool of pre-registered panelists rather than a defined sampling frame.

Because participants were recruited from an online panel, the sample may overrepresent individuals with higher digital literacy and stable internet access, and may not fully represent all Japanese working women aged 40–60 years. In addition, participation was voluntary, which may have introduced self-selection bias. These limitations should be considered when interpreting the findings, particularly with regard to generalizability.

The study protocol was approved by the Institutional Ethics Committee of Showa Medical University (Approval No. 2023-299-A). This study was conducted as part of the project “*Evidence Development of the Simplified Menopausal Index (SMI) and Its Association with Work Productivity*,” funded by the Japan Agency for Medical Research and Development (AMED) under the program “*Establishing R&D Infrastructure toward Social Implementation of Prevention and Health Promotion*”.

### 2.2. Survey Measures

Three primary constructs were assessed in this study:

(1) Menopausal symptoms;
(2) Work productivity;
(3) Workplace openness.

#### 2.2.1. Menopausal Symptoms

Menopausal symptoms were assessed using the Simplified Menopause Index (SMI), a self-administered scale developed in Japan to evaluate the severity of climacteric symptoms [10,11]. The SMI consists of 10 items covering both physical and psychological symptoms, categorized into three domains:

- Vasomotor (e.g., hot flashes, sweating);
- Psychological (e.g., irritability, anxiety, insomnia, depressed mood);
- Somatic (e.g., shoulder stiffness, joint pain).

Participants rated each symptom using four response categories (“strong,” “moderate,” “mild,” “none”). Each item has a predefined scoring weight, and the total SMI score is calculated by summing the weighted values of all items. Higher scores indicate more severe menopausal symptoms. As a general guideline, a total score of ≥51 suggests the need for medical consultation. A full list of SMI items and domain classifications is provided in the Appendix A.

#### 2.2.2. Work Productivity

Work productivity was measured using the World Health Organization’s Health and Work Performance Questionnaire (HPQ) [12], a widely used and psychometrically validated instrument for assessing self-rated work productivity, absenteeism, and presenteeism. The HPQ has established reliability and validity internationally [13], and its Japanese version has demonstrated satisfactory construct validity and test–retest reliability [14].

The HPQ includes two indices of presenteeism:

- Absolute presenteeism;
- Relative presenteeism.

The present study used absolute presenteeism, which ranges from 0 to 10, with higher values indicating better work performance (higher productivity). Productivity loss can be estimated as 100%−(absolute presenteeism × 10). A previous Japanese prospective study suggested that an absolute presenteeism score < 4.0 may indicate elevated risk of mental sickness absence [15]. HPQ scores reflect productivity during the previous four weeks.

#### 2.2.3. Workplace Openness

Workplace openness was assessed using a single item asking whether participants felt comfortable discussing health concerns, including menopausal symptoms, with supervisors or colleagues. Responses were categorized as “yes,” “no,” and “not sure” (coded as 1, 2, and 3, respectively) and treated as categorical variables in the regression analysis. This single-item approach was adopted to minimize respondent burden and ensure feasibility in a large-scale nationwide survey, while capturing participants’ overall perceptions of openness in their workplace.

Covariates included age, educational attainment, marital status, living with children, employment status, daily working hours, and years of service.

### 2.3. Statistical Analysis

Covariates were selected a priori based on previous literature on work productivity and menopausal symptoms, as well as data availability within the survey. These included age, marital status, educational attainment, employment characteristics, and working hours, which have been consistently associated with work productivity in occupational health research. Variables such as detailed job strain measures or workplace size were not available in the present dataset and therefore could not be included.

Analyses were conducted using complete-case data. The proportion of missing data for key variables was low, and no systematic patterns of missingness were observed.

Multiple linear regression analyses were conducted to examine the associations between menopausal symptoms (total SMI score and symptom domains) and self-rated work productivity. Standardized beta coefficients (β) were estimated. Categorical variables were dummy-coded with the following reference groups:

- Age: 40–44 years;
- Education: junior high school/high school graduate;
- Marital status: never married;
- Living with children: yes;
- Employment status: managerial;
- Workplace openness: yes.

Two primary models were fitted:

- Model 1: total SMI score;
- Model 2: the three SMI domains (vasomotor, psychological, somatic), simultaneously included to estimate domain-specific associations while adjusting for the same covariates
- Domain-specific single-predictor models (Models 3–5) were additionally examined as sensitivity analyses and are presented in the Supplement to illustrate marginal associations.

To assess potential multicollinearity among the three SMI domains included simultaneously in the regression model, variance inflation factors (VIFs) were calculated and are reported in Table A1.

Statistical analyses were conducted using IBM SPSS Statistics Version 28.0 (IBM Corp., Armonk, NY, USA). Statistical significance was set at *p* < 0.05.

## 3. Results

### 3.1. Demographic Statistics of the Survey

Among the 4000 respondents, the largest age group was women aged 50–54 years (29.1%), followed by those aged 45–49 years (27.0%). Most participants were university graduates (31.2%), while 30.5% were junior high school or high school graduates, and 21.3% were junior college graduates. More than half (56.8%) were married or living with a partner, and 40.3% lived with children. Only 4.4% held managerial positions, whereas the majority (95.6%) were general staff. Participants reported an average of 6.8 working hours per day (SD = 2.2) and an average of 9.9 years of service (SD = 8.6).

Regarding workplace openness, one in four participants (25.0%) felt able to discuss menopausal health concerns at work, while 44.1% answered “no” and 31.0% were “not sure.” The mean absolute presenteeism (work productivity) score was 5.7 (SD = 2.1), corresponding to an estimated 43% productivity loss compared with each respondent’s self-rated best productivity. The mean total SMI score was 34.9 (SD = 21.8). By domain, mean scores were 12.7 (SD = 9.9) for vasomotor symptoms, 15.2 (SD = 10.6) for psychological symptoms, and 7.0 (SD = 4.3) for somatic symptoms (Table 1).

### 3.2. Analytical Overview

To examine the associations between menopausal symptoms and work productivity, we conducted two multiple linear regression models.

- Model 1 evaluated the association between the total SMI score and self-rated work productivity.
- Model 2 simultaneously included the three SMI domains (vasomotor, psychological, and somatic symptoms) to identify domain-specific effects while adjusting for the same covariates.

By comparing these two models, we aimed to clarify which aspects of menopausal symptoms most strongly influence productivity. Details of the SMI domains and item composition are provided in Table A1.

### 3.3. Model 1: Association Between Total SMI Score and Work Productivity

Multiple linear regression analysis was performed to examine the association between overall menopausal symptom severity and self-rated work productivity (HPQ), adjusting for age, education, marital status, living with children, employment status, daily working hours, years of service, and workplace openness (Table 2).

After adjustment, higher SMI total scores were significantly associated with lower work productivity (β = −0.146, *p* < 0.001). Among covariates, being in one’s 50 s (vs. 40 s), higher educational attainment, being married or divorced, longer working hours, and longer years of service were positively associated with productivity. Conversely, participants who reported that their workplace was “not open” (β = −0.162, *p* < 0.001) or “not sure” (β = −0.089, *p* < 0.001) about discussing menopause had lower HPQ scores.

### 3.4. Model 2: Association Between SMI Domain Scores and Work Productivity

To further clarify which aspects of menopausal symptoms most strongly influence productivity, a second regression model was conducted using the three SMI domain scores (vasomotor, psychological, and somatic) instead of the total score, while maintaining the same covariates as in Model 1. This approach allowed identification of domain-specific effects on self-rated work productivity.

**Figure 2:**
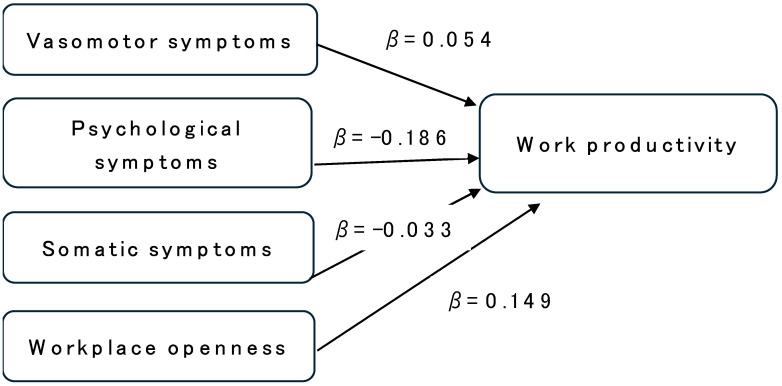
Standardized regression coefficients (β) for factors associated with self-rated work productivity (HPQ). Model 2 includes the three SMI domains (vasomotor, psychological, and somatic symptoms) and covariates. Psychological symptoms and lower workplace openness were significantly associated with reduced productivity. Error bars represent 95% confidence intervals.

Table 2 presents the associations between the total SMI score, the three SMI domains, and self-rated work productivity (HPQ). After adjustment for covariates, psychological symptoms showed the strongest negative association (β = −0.186, *p* < 0.001). Vasomotor symptoms showed a small positive coefficient (β = 0.054, *p* = 0.007). Somatic symptoms were not significantly associated with productivity (β = −0.033, *p* = 0.121) (Figure 2). Among covariates, being in one’s 50 s (vs. 40 s), higher educational attainment, being married or divorced, longer working hours, longer years of service, and supportive workplace communication were significantly associated with higher productivity.

In the model including all three SMI domains simultaneously, variance inflation factors ranged from 1.79 to 2.16, indicating no evidence of severe multicollinearity (Table A1).

Figure 2 illustrates the conceptual framework of the multiple regression model examining the associations between menopausal symptom domains, workplace openness, and work productivity. Arrows indicate the direction of the associations, with standardized coefficients (β) displayed for each predictor. Psychological symptoms showed the strongest negative association with productivity, whereas vasomotor symptoms showed a small positive coefficient. Workplace openness demonstrated a consistent positive relationship with work productivity.

## 4. Discussion

This study examined cross-sectional baseline associations between menopausal symptoms, workplace openness, and self-rated work productivity among a large sample of Japanese working women participating in a prospective cohort study.

While menopausal symptoms have traditionally been regarded as individual health concerns, our findings indicate that workplace environments—particularly psychological safety and openness—are closely related to productivity. These findings reinforce the importance of organizational support for women’s midlife health [1,2,8] and clarify that psychological symptoms exert a stronger impact on work productivity compared with vasomotor or somatic symptoms.

Workplace openness to discussing menopausal symptoms showed a strong and consistent association with productivity across all models. This pattern aligns with previous research demonstrating that organizational culture, communication norms, and psychological safety influence both employee well-being and productivity [8,9]. Women who perceived their workplace as “not open” or “uncertain” reported lower productivity, suggesting that organizational climate may either buffer or exacerbate symptom-related challenges. These findings emphasize the importance of cultivating psychologically safe environments that facilitate open dialogue and access to consultation, enabling timely support and flexible accommodations to sustain productivity during health challenges such as menopause.

Psychological symptoms demonstrated the strongest negative association with self-rated work productivity. Consistent with the present findings, earlier reports have shown that irritability, anxiety, sleep disturbance, and low mood impair concentration, decision-making, and interpersonal communication in workplace settings [6,7]. For working women, maintaining good mental health is essential to sustaining daily work productivity, particularly for those in managerial or leadership roles. Because many midlife women occupy coordinating or supervisory positions, psychological burden may directly translate into reduced efficiency in daily work. This suggests that psychological and emotional support should be central components of workplace health initiatives for midlife women.

When the three SMI domains were included simultaneously in the regression model, a small positive coefficient was observed for vasomotor symptoms. Correlation analyses indicated moderate correlations among the SMI domains, and variance inflation factors were below commonly used thresholds, suggesting the absence of severe multicollinearity. This pattern is consistent with a potential suppression effect arising from shared variance among symptom domains, although this interpretation should be made with caution. Although several associations reached statistical significance, the magnitude of some regression coefficients was small. Given the large sample size, these findings should be interpreted primarily in terms of patterns and directions of association rather than large individual-level effects. From a public health perspective, even modest associations may be meaningful when they affect a large population of working women.

Vasomotor symptoms such as hot flashes or palpitations are often episodic and may be less disruptive to sustained work tasks than psychological or somatic symptoms [4,7]. However, prior research, including Coronado et al. [16], has reported that vasomotor symptoms can impair work productivity and daily living. Further item-level analyses are warranted to examine how specific symptom types and their frequency or severity influence productivity.

Somatic symptoms were not significantly related to productivity, possibly because self-management or workplace adaptations mitigate their effects. Physical discomfort, such as stiffness or pain, can interfere with task performance but may be alleviated through pacing or ergonomic accommodations [7]. O’Neill et al. [17] similarly reported that psychological and neurocognitive symptoms, rather than somatic ones, showed stronger associations with work outcomes. Future research should explore specific somatic symptoms in greater detail.

Beyond menopausal symptoms, several background variables were also associated with work productivity. Women aged 50–59 showed higher productivity than those in their 40 s, likely reflecting greater job experience, role adaptation, or reduced symptom severity after menopause. Higher educational attainment was linked to higher productivity, potentially reflecting greater health literacy and access to professional roles that allow flexibility and autonomy. Longer working hours and years of service were also associated with higher self-rated productivity, which may indicate enhanced job familiarity and perceived competence. Married women or those living with partners reported higher productivity, possibly due to emotional or practical support outside the workplace. These findings suggest that productivity during the menopausal transition is influenced by a complex interplay of individual, occupational, and social factors.

Overall, these findings underscore that many midlife women experiencing menopausal symptoms are in positions of responsibility, managing both professional and domestic roles. Given individual variability in symptom severity, it is important that women recognize and address their symptoms through appropriate consultation and workplace collaboration. The current results highlight the importance of both self-awareness and organizational awareness of menopausal health, consistent with Japan’s *Health and Productivity Management (HPM)* framework. As more women remain active in the workforce through midlife, menopausal health should be recognized not only as a personal issue but as part of broader corporate well-being strategies. Creating psychologically safe and inclusive workplaces that support health-related discussions may sustain productivity and promote long-term employee retention.

Notably, the association between workplace openness and presenteeism may be bidirectional. Employees with lower productivity or poorer health may hesitate to disclose symptoms, further reducing psychological safety and perpetuating reduced productivity. Future longitudinal research using subsequent survey waves is warranted to clarify causal directions and mediating mechanisms. Given cultural similarities across Asian workplaces, the SMI may serve as a practical assessment tool in settings beyond Japan to support midlife women’s health and work engagement. While this study focused on Japanese working women, the broader implications may extend to other contexts with similar workplace cultures, particularly where menopause remains a sensitive or under-discussed topic. At the same time, differences in labor systems, gender norms, and occupational health frameworks should be carefully considered when applying these findings to other settings.

From an occupational and public health perspective, the findings highlight the importance of considering both individual health factors and workplace context when addressing productivity among midlife working women. Within these limitations, the observed associations suggest that menopausal symptoms and perceptions of workplace openness tend to co-occur in ways that may be relevant to work functioning.

These results underscore the potential relevance of organizational approaches that foster psychological safety, provide opportunities for consultation, and normalize discussions around women’s health in the workplace. Such approaches may complement individual-level health care and contribute to more supportive work environments for midlife women. Importantly, the findings may help organizations identify areas where preventive or supportive interventions could be prioritized, even before causal pathways are fully established.

### Limitations of the Study

This study has several limitations. First, because the analysis was based on cross-sectional baseline data, the observed associations cannot establish temporal order or causality. In particular, it is plausible that reduced work productivity may influence individuals’ perceptions of workplace openness, rather than workplace openness influencing productivity. This possibility of reverse causality should be considered when interpreting the findings. Second, all measures—including the SMI, HPQ, and workplace openness—were self-reported, which may introduce reporting or recall bias. In addition, workplace openness was assessed using a single-item measure, which captures participants’ perceived openness to discussing health concerns but may not fully reflect broader constructs such as psychological safety or organizational climate. Third, although the sample was large and diverse, participants were limited to Japanese working women recruited via an online panel, which may restrict generalizability. Finally, while the SMI is widely used in Japan, its psychometric properties require further validation for occupational health research.

Future longitudinal analyses using multi-wave data from this cohort will help confirm the observed associations and explore mediating or moderating factors such as organizational support, job type, and coping strategies.

## 5. Conclusions

This study used baseline data from a large-scale cohort survey to examine how menopausal symptoms and workplace openness relate to work productivity among Japanese working women. When the three symptom domains were analyzed simultaneously, psychological symptoms showed the strongest negative association with productivity, while vasomotor symptoms displayed a small positive coefficient. Somatic symptoms were not significantly associated. Importantly, workplace openness to discussing health concerns was consistently linked with higher productivity, suggesting that supportive and psychologically safe organizational climates may buffer the adverse effects of menopausal symptoms.

Future longitudinal analyses using follow-up data will help clarify causal pathways and inform interventions to sustain productivity and promote well-being among midlife women in the workforce.

## Figures and Tables

**Table 1:** Descriptive statistics of study variables (N = 4000). Demographic and occupational characteristics, menopausal symptom scores (total and by domain), workplace openness, and self-rated work productivity (HPQ) among Japanese working women. Values are presented as mean (SD) for continuous variables and *n* (%) for categorical variables.

Characteristics		*n* (%)
Age	40–44 years old	913 (22.8)
	45–49 years old	1081 (27.0)
	50–54 years old	1163 (29.1)
	55–59 years old	722 (18.1)
	60 years old	121 (3.0)
Education	Junior high school/high school graduate	1218 (30.5)
	Vocational school graduate	578 (14.5)
	Junior college graduate	854 (21.3)
	University graduate	1249 (31.2)
	Graduate school graduate	101 (2.5)
Marital status	Never married	1168 (29.2)
	Married or living with partner	2273 (56.8)
	Widowed	51 (1.3)
	Divorced/Separated	508 (12.7)
Living with children	Yes	1612 (40.3)
	No	2388 (59.7)
Employment status	Managerial positions	176 (4.4)
	General positions	3824 (95.6)
Daily working hours		6.78 (mean) 2.2 (SD)
Years of service		9.9 (mean) 8.6 (SD)
Workplace openness to discussing menopause	Yes	999 (25.0)
	No	1763 (44.1)
	Not sure	1238 (31.0)
WHO-HPQ (presenteeism score)		5.7 (mean) 2.1 (SD)
SMI (Simplified Menopausal Index)	Total score	34.9 (mean) 21.8 (SD)
SMI (domains)	Vasomotor symptoms score	12.69 (mean) 9.9 (SD)
	Psychological symptoms score	15.18 (mean) 10.61 (SD)
	Somatic symptoms score	6.99 (mean) 4.25 (SD)

**Table 2:** Comparison of two multivariable regression models evaluating the associations between total SMI score, SMI domain scores, and self-rated work productivity. Model 1 includes the total SMI score, and Model 2 includes the three SMI domains. All models adjust for age, education, marital status, living with children, employment status, daily working hours, years of service, and workplace openness to discussing menopause. Standardized coefficients (β) are shown.

Characteristics		Model 1 β (SMI Total)	Model 1 *p*	Model 2 β (SMI Domains)	Model 2 *p*
Age	45–49 (ref: 40–44)	0.003	0.885	0.003	0.858
	50–54	0.057	0.004 *	0.053	0.007 **
	55–59	0.097	<0.001 ***	0.091	<0.001 ***
	60	0.019	0.240	0.019	0.243
Education	Vocational school graduate (ref: Junior high/high school graduate)	0.029	0.093	0.031	0.071
	Junior college graduate	0.054	0.002 **	0.054	0.002 **
	University graduate	0.083	<0.001 ***	0.084	<0.001 ***
	Graduate school graduate	0.042	0.008 **	0.043	0.006 **
Marital status	Married or living with partner (ref: never married)	0.089	<0.001 ***	0.086	<0.001 ***
	Widowed	0.015	0.354	0.014	0.364
	Divorced/Separated	0.042	0.022 *	0.040	0.029 *
Living with children	No (ref: Yes)	0.015	0.408	0.019	0.283
Employment status	General (ref: Managerial)	0.024	0.123	0.020	0.208
Daily working hours	NA	0.099	<0.001 ***	0.098	<0.001 ***
Years of service	NA	0.053	<0.001 ***	0.051	0.001 **
Workplace openness to discussing menopause	No (ref: Yes)	−0.162	<0.001 ***	−0.149	<0.001 ***
	Not sure	−0.089	<0.001 ***	−0.082	<0.001 ***
SMI score	Total	−0.146	<0.001***	−0.079	<0.001 ***
	Vasomotor symptom domain	NA	NA	0.054	0.007 **
	Psychological symptom domain	NA	NA	−0.186	<0.001 ***
	Somatic symptom domain	NA	NA	−0.033	0.121

* *p* < 0.05, ** *p* < 0.01, *** *p* < 0.001.

## Data Availability

The data presented in this study are available from the corresponding author on reasonable request. The data are not publicly available due to ethical restrictions.

## Appendix A. English Translation of the Simplified Menopause Index (SMI) Items

1. Hot flashes
2. Excessive sweating
3. Sensitivity to cold in the lower back or extremities
4. Shortness of breath or palpitations
5. Difficulty falling asleep or light sleep
6. Irritability or nervousness
7. Mood swings or depressive feelings
8. Frequent headaches, dizziness, or nausea
9. Fatigability or easy exhaustion
10. Stiff shoulders, back pain, or pain in the limbs

(Adapted from Koyama et al., 1994, Simplified Menopause Index, [10])

**Table A1:** Variance inflation factors (VIFs) for the three SMI domains in the simultaneous domain model. All VIF values were below commonly used thresholds, indicating no evidence of severe multicollinearity.

Predictor (SMI) Domain	VIF
Vasomotor symptoms	1.79
Psychological symptoms	2.16
Somatic symptoms	2.02

## Appendix B. Domain-Specific Regression Models (Models 3–5)

Model 3: Association between Vasomotor Symptoms and Work Productivity.

In the domain-specific model, vasomotor symptoms were significantly associated with work productivity (β = −0.079, *p* < 0.001). However, this association was attenuated when all symptom domains were included simultaneously. Although statistically significant, the effect size was modest, indicating that the association between vasomotor symptoms and work productivity was weaker than that observed for psychological symptoms.

Model 4: Association between Psychological Symptoms and Work Productivity.

Psychological symptoms showed a substantially stronger association with work productivity (β = −0.174, *p* < 0.001), indicating strong associations with reduced concentration, communication, and motivation at work. Among the domain-specific models, psychological burden exerted the greatest impact on work productivity.

Model 5: Association between Somatic Symptoms and Work Productivity.

Somatic symptoms were significantly associated with lower productivity (β = −0.126, *p* < 0.001), although the magnitude of the association was smaller than that observed for psychological symptoms.

### Summary of Domain-Specific Results

Overall, psychological symptoms had the strongest detrimental impact on work productivity, followed by a smaller effect of somatic symptoms, whereas vasomotor symptoms showed a small coefficient in the simultaneous model.

These results suggest that the psychological dimension of menopausal symptoms plays a central role in productivity during midlife (Table A2).

**Table A2:** Comparison of Three Multivariable Regression Models (Models 3–5). * Comparison of multivariable regression models evaluating the associations between different menopausal symptom domains and self-perceived work productivity. All models adjust for age, education, marital status, living with children, employment status, daily working hours, years of service, and workplace openness to discussing menopause. Standardized coefficients (β) are presented.

Characteristics		Model 3 β (Vasomotor)	Model 3 *p*	Model 4 β (Psychological)	Model 4 *p*	Model 5 β (Somatic)	Model 5 *p*
Age	45–49 (ref: 40–44)	0.001	0.961	0.002	0.917	0.007	0.735
	50–54	0.056	0.005 **	0.053	0.007 **	0.062	0.002 **
	55–59	0.102	<0.001 ***	0.091	<0.001 ***	0.103	<0.001 ***
	60	0.022	0.189	0.018	0.276	0.024	0.148
Education	Vocational school graduate (ref: Junior high/high school graduate)	0.029	0.095	0.030	0.084	0.031	0.070
	Junior college graduate	0.057	0.002 **	0.053	0.003 **	0.057	0.001 ***
	University graduate	0.088	<0.001 ***	0.083	<0.001 ***	0.088	<0.001 ***
	Graduate school graduate	0.042	0.008 **	0.042	0.007 **	0.043	0.006 **
Marital status	Married or living with partner (ref: never married)	0.090	<0.001 ***	0.088	<0.001 ***	0.087	<0.001 ***
	Widowed	0.015	0.350	0.015	0.343	0.013	0.411
	Divorced/Separated	0.043	0.020 *	0.041	0.024 *	0.040	0.030 *
Living with children	No (ref: Yes)	0.012	0.523	0.017	0.348	0.019	0.308
Employment status	General (ref: Managerial)	0.026	0.098	0.021	0.184	0.025	0.115
Daily working hours	NA	0.099	<0.001 ***	0.097	0.001 **	0.103	<0.001 ***
Years of service	NA	0.056	<0.001 ***	0.052	0.001 **	0.052	0.001 **
Workplace openness to discussing menopause	No (ref: Yes)	−0.178	<0.001 ***	−0.152	<0.001 ***	−0.167	<0.001 ***
	Not sure	−0.091	<0.001 ***	−0.085	<0.001 ***	−0.088	<0.001 ***
SMI score	NA	−0.079	<0.001 ***	−0.174	<0.001 ***	−0.126	<0.001 ***

* *p* < 0.05, ** *p* < 0.01, *** *p* < 0.001.
